# Automotive workers: the role of coordinative and conditional abilities as effectiveness wellness indicator

**DOI:** 10.3389/fpubh.2024.1447358

**Published:** 2024-10-14

**Authors:** Angelo Rodio, Tommaso Di Libero, Alessandro Biffi, Fredrick Fernando, Luigi Fattorini

**Affiliations:** ^1^Department of Human Sciences, Society and Health, Sustainable Living Concept Xlab Marco Marchetti, Via Sant’Angelo in Theodice Campus Folcara, University of Cassino and Southern Lazio, Cassino, Italy; ^2^Med-EX, Medicine & Exercise, Medical Partner Scuderia Ferrari, Rome, Italy; ^3^Department of Physiology and Pharmacology “Vittorio Erspamer”, Sapienza University of Rome, Rome, Italy

**Keywords:** corporate wellness, occupational position, physical activity, motor abilities, handgrip, flexibility, reaction time

## Abstract

**Introduction:**

Noncommunicable diseases are on the rise due to sedentary and unhealthy lifestyles. The World Health Organization (WHO) and the American College of Sports Medicine (ACSM) recommend maintaining a healthy diet and engaging in regular physical activity, particularly in the workplace. Prolonged and repetitive work tasks can result in extended sick leave and decreased productivity while at work. Therefore, it is important to identify predictive factors that can act as indicators of the health status of employees. Utilizing motor abilities assessment tests can help to identify health issues at an early stage. Promoting preventive health measures is crucial for addressing chronic diseases and enhancing overall occupational well-being. The purpose of the study was to characterize workers’ motor abilities and fitness levels and identify potential indicators.

**Methods:**

A total of 605 workers participated in this study, including 529 male participants with a height of 176 ± 0.09 cm and a body mass of 75.9 ± 14.1 kg and 77 female participants with a height of 162 ± 0.11 cm and an average body mass of 58.9 ± 11.1 kg. These individuals were enrolled during an Italian automotive corporation’s mandatory annual medical check-up. The participants were categorized into three groups based on their occupational roles: blue-collar, manager, and white-collar groups. The participants underwent motor abilities assessment tests for upper-limb strength, trunk flexibility, and reaction time.

**Results:**

The blue-collar group reported the best results in upper-limb strength (93.3 kgf ± 18.60), but had the worst results in flexibility (21.7 cm ± 7.90), total reaction time (58.8 s ± 4.74); and average intermedium (0.68 ms ± 0.11). The white-collar group reported the best result in flexibility (23.7 cm ± 8.94) and reaction time (48.5 s ± 4.38; 0.64 ms ± 0.09).

**Conclusion:**

Despite being frequently used to assess health status, handgrip measurements may not provide accurate differentiations because of the common use of blue-collar workers for tasks that require upper-limb strength. In contrast, reaction time metrics appear more reliable and discriminative in evaluating a worker’s physical fitness.

## 1 Introduction

A significant number of non-communicable diseases, often linked to sedentary and unhealthy lifestyles, are prevalent worldwide, especially in high-income countries (1). Urbanization, technological advancements, and globalization have led to an energy imbalance due to decreased physical activity, precipitating a heightened risk of acute and chronic diseases (2). According to the scientific community, key risk factors and indicators for non-communicable diseases, such as cardiovascular and respiratory conditions, metabolic disorders, certain types of cancer, and obesity, are correlated with factors including high waist circumference, dyslipidemia, hypertension, insulin resistance, and physical inactivity. Notably, obesity has been recently classified as a disease by the World Health Organization (WHO), designated with code 5B81 in the WHO International Classification of Diseases (3–5). Furthermore, the presence of these indicators in apparently healthy individuals is termed Metabolic Syndrome (MS) (6). MS is defined as an altered clinical condition that is not categorized as a disease but as a physical state that could progress into acute or chronic pathologies (7). In this regard, although individuals with chronic illnesses have a life expectancy similar to that of healthy individuals, their physical condition necessitates ongoing care services. This diminishes their quality of life and leads to increased public health expenditure (8). It is imperative to implement virtuous strategies to enhance the quality of life (8). The WHO and the American College of Sports Medicine (ACSM) have established guidelines endorsing the correlation between a healthy diet and increased physical activity in effectively mitigating risk factors (9). These preventive measures should be seamlessly integrated into daily routines to improve compliance among individuals (10). Implementing intervention programs within the workplace is recommended, particularly for employees who spend most of their work hours sitting (11). In certain occupational tasks, workers may engage in physical activities that align with the ACSM recommendations (12, 13). However, Andersen et al. (14) found that long-term sickness absence (LTSA) can be linked to specific repetitive movements in the workplace, such as neck flexion, hand/arm repetition, working while bending forward, working with a bent or rotated back and elevated arms, as well as vibration and heavy lifting (14–16). Moreover, some studies have indicated that workers who engage in physical activities during work hours may be reluctant to participate in planned physical activities outside of work (17, 18). It is essential to consider the type of physical activity to effectively achieve the goal of prevention (19). In addition, certain motor impairments do not necessarily result in temporary functional limitations that require long-term sick leave. This is known as presenteeism and can lead to reduced productivity (20). This may contribute to an increase in work-related accidents and a decline in overall work performance. In Italy, the protection of workers’ health and safety is regulated by Legislative Decree No. 81 (D.lgs. 9 April 2008) Franco and Mora (21), which outlines practical interventions to ensure workplace health and safety. This includes the importance of preventive and periodic health examinations aimed at safeguarding workers’ health and well-being in relation to specific occupational risk factors. In addition to health status, motor functional abilities play a significant role in assessing the risk of illness to minimize presenteeism and long-term sick leave (22). Motor abilities are categorized as coordinative and conditional, with the former encompassing balance, flexibility, rhythm, and reaction, while the latter encompasses strength, speed, and endurance. Aerobic power and muscular strength have been well-documented in scientific literature as positively impacting quality of life. Similarly, maintaining good flexibility has been associated with a decreased risk of low back injuries and reduced perceived pain, thereby improving movement capabilities (23). In addition, studies have shown that coordinative abilities, balance, and reaction time play a significant role overall (24, 25). The primary objective of the current study was to evaluate the coordinative and conditional abilities of a large sample of workers as a means of assessing their fitness levels and overall well-being (26, 27). Longitudinal monitoring of these parameters could potentially serve as predictive indicators for occupational physicians when tailoring physical activity recommendations (28, 29). Furthermore, such evaluations could contribute to the design of specific physical activity interventions as targeted preventative measures. In pursuit of our objectives, we chose to focus on the automotive sector because of its representation of various occupational positions (OPs), including managers, white-collar workers, and blue-collar workers (30). In addition to assessing health status, it is essential to consider motor functional abilities when evaluating the risk of illness to reduce presenteeism and long-term sickness absence (LTSA) (22). Motor abilities are typically categorized as coordinative and conditional. Coordinative abilities encompass balance, flexibility, rhythm, and reaction, while conditional abilities involve force, velocity, and resistance (31, 32). It is worth noting that evaluating an individual’s motor skills can also help customize physical activity interventions to align with the ACSM recommendations (33). Existing scientific literature strongly supports the positive correlation between aerobic power, muscular strength, and quality of life. In addition, optimal flexibility is associated with a decreased likelihood of low back injuries and reduced perceived pain that may limit movement capabilities (23). Furthermore, coordinative abilities, such as balance and reaction time, play a significant role in overall wellness (24, 25). This study aimed to assess the coordinative and conditional abilities of a large sample of workers to assess their fitness levels as indicators of well-being and health (26, 27). Longitudinal monitoring of these parameters can also serve as predictive tools for occupational physicians when tailoring physical activity recommendations (28, 29). Moreover, such evaluations can be valuable in designing specific physical activity interventions as targeted preventive measures. For this study, the automotive sector was chosen due to the diverse occupational positions, ranging from managers and white-collar workers in office-based roles to blue-collar workers performing more physically demanding tasks (30).

## 2 Materials and methods

### 2.1 Subjects

A large number of workers (*N* = 605; 529 male participants: height, 1.76 ± 0.09 m and body mass, 75.9 ± 14.1 kg; and 77 female participants: height, 1.62 ± 0.11 m and body mass, 58.9 ± 11.1 kg) voluntarily agreed to participate in this study. They were enrolled during their mandatory annual medical examination at an Italian automotive corporation. Written informed consent was obtained from each participant after a detailed explanation of the test protocol and before starting the experimental procedures. The study was in accordance with the Declaration of Helsinki, and no participant received any compensation. As mentioned by Fukushima (30), all participants were divided into three groups based on their occupational position within the corporation (30). Specifically, for conducting these evaluations, the participants were then divided into the following groups: MNG, which included managers and corporate executives; WCG, which included office-based workers performing different tasks; and BCG, which included construction workers or other professionals engaged in manual or practical work. MNG workers are primarily involved in strategic planning, supervision, and decision-making activities. They invest most of their time in meetings, working on computers, or handling other administrative tasks. WCG workers are predominantly engaged in administrative, accounting, planning, or technical support activities. Their work is largely sedentary, involving extensive computer use, and may include brief interdepartmental or interoffice travel. On the other hand, BCG workers are directly immersed in production, assembly, and maintenance tasks. Their work is physically demanding, requiring strength, coordination, and endurance as they lift weights, stand for extended periods, and carry out repetitive movements.

### 2.2 Experimental protocol

**Figure 1:**
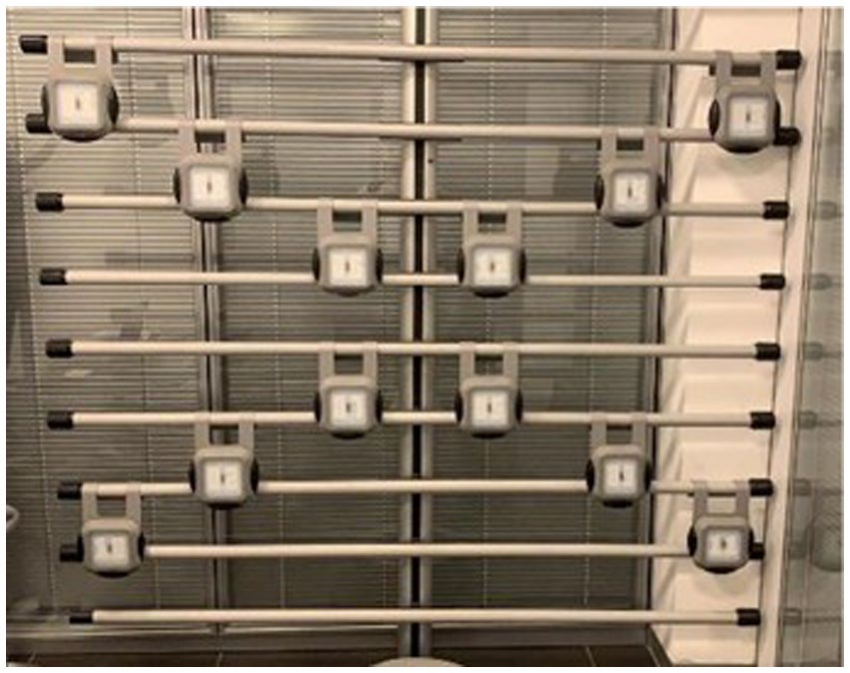
Reaction time satellite system.

The protocol consisted of a battery test to assess the coordinative and conditional abilities. To evaluate the participants’ upper limb strength and trunk flexibility, the handgrip (HG) test (34, 35) and the sit-and-reach (SR) (36) test were used, respectively; while the oculo-manual test was used to evaluate the coordinative abilities by measuring the reaction time. Specifically, the HG test allows for the indirect evaluation of the functional profile by assessing the contractile force of the hand flexor muscles using a dynamometer (HandgripDynX®, AKERN SRL, FI, Italia). The participants held the dynamometer in the hand being tested, with the arm at a right angle and the elbow at the side of the body. Avoiding anybody other body movements, they were then instructed to squeeze the dynamometer with maximum isometric effort and maintain this position for about 5 s. They were also asked to take 30 s of rest between each test. Both the dominant and non-dominant sides were evaluated, and the best of three repetitions for each arm was considered. At the end of the test, the sum of the best performances from the dominant and non-dominant sides was computed. The YMCA SR test measures the flexibility of the posterior chain, including the lower back and hamstring muscles. Before testing, the participants were allowed to stretch and perform moderate aerobic exercise as a warm-up. Afterward, they sat on the floor without shoes and heels, bisecting the yardstick. The hands were placed on top of each other in front of the participant. The participants were asked to slowly bend forward, maintaining the extension of the knees, and touch the yardstick for about 2 s to maintain the distance longer. The better of the two repetitions was recorded. Finally, the workers completed an oculo-manual reaction test. The device consists of 12 satellite light sensors fixed at eye level and arranged as shown in Figure 1. They light up alternately in a random order for a total of 54 times. Before the test, the participants were positioned in front of the lighting sensor grid at a comfortable distance to touch the four corners with their feet. The participants, while keeping their posture erect in front of the device, touched the illuminated sensor as quickly as possible using their hands. The next light turns on only after the current light is switched off. The reaction time was calculated as the total time (RT-TT) taken in seconds to turn off all 54 lights and in terms of the average intermedium (RT-AI) reaction time. The best performance from three repetitions was used in the subsequent statistical analysis. To the best of our knowledge, regarding reaction time parameters, reference tables that take sex and age into account were not found in the literature. Thus, it was impossible to rank the participants’ performance; instead, we focused on the absolute values, as done in previous work by Di Libero et al. (37).

## 3 Statistical analysis

For the statistical analysis, IBM® SPSS® Statistics 25.0 (SPSS, IBM, USA) software was used, with a significance level set at *p* = 0.05. An observational study was conducted on all samples of the 605 workers to analyze the frequencies and the percentages, stratifying them by activity and sex. Descriptive statistics were performed on the anthropometric data, with mean and standard deviation values (or median and interquartile range values) computed to characterize all workers and stratify them by sex. The Shapiro–Wilk/Kolmogorov–Smirnov normality test was performed to assess the normality distribution of all variables in both male and female participants, dividing subjects in the three groups by activity (MNG, WCG, and BCG). The anthropometric and performance variables were categorized by sex and occupational position (MNG, BCG, and WCG). According to the result of the normality test, the two-tailed Pearson or the Spearman correlation analysis was conducted. Separating the male and female participants, if the measured variables were normally distributed within the three groups by activity, a One-way ANOVA was performed, and, if it was significant (*p* ≤ 0.05), a *post hoc* test using the Bonferroni correction (*p* ≤ 0.0175) was performed. This was done to investigate in which pairwise comparisons of the groups by activity the significant differences lay. If the normality test was not satisfied, the corresponding non-parametric analyses, including the Kruskal–Wallis and Mann–Whitney U tests for all pairwise comparisons, were performed. Due to the fixed position of the satellite lights, the test was considered height-dependent. Therefore, further analysis was conducted including only participants belonging to a specific range characterized by height and occupational position.

## 4 Results

The total sample consisted of 50% BCG, 40% WCG, and 10% MNG. The relative percentages of the participants, divided by sex and OP, are shown in Table 1. In Table 2, the anagraphic and anthropometric data are reported and broken down by sex and OP. In addition, as shown in Table 2, it was observed that the male participants were characterized by qualitative classification, with the BCG and MNG falling into the slightly overweight category [body mass index (BMI) ranging from 25 and 29.9], while the WCG fell within the normal weight category (BMI ranging from to 18.5 and 24.9). In addition, in the all-female groups, the BMI was at a normal weight level. Table 3 presents information on the physical activity performed outside the workplace. The activities primarily included indoor and outdoor sports, such as running, swimming, cycling, hiking, futsal, and gym workouts, with a focus on weekly participation. As shown in this table, it was evident that the MNG and WCG were the most active compared to the BCG, for both male and female participants, with the female participants in the MNG having the highest percentage of activity. The coordinative and conditional abilities results are shown in Table 4. Regarding the test results, the only ones with a validated reference table for comparing absolute values were the SR Bhutta et al. (36) and HG Thompson et al. (38). Regarding handgrip sum (HGsum), the participants were placed at a regular level, with scores ranging between 86 and 115 kg for the male participants and very poor for the female participants, with scores lower than 71 kg for all three groups investigated. Regarding flexibility, in the BCG and WCG, both sexes reported scores well above the average level (scores ranging from 21 and 22 cm). In addition, the scores of the male participants in the MNG were well above the average level, while an above-average level was found for the female participants (scores ranging from 19 and 21 cm). For RT-TT, the three groups varied between 47 s and 50s for the male participants and between 52 s and 58 s for the female participants. The RT-AI results ranged between 0.62 ms and 0.67 ms for the male participants and between 0.71 ms and 0.83 ms for the female participants. In both reaction time parameters, the female participants performed less well than the male participants. In all tests assessing the coordinative and conditional abilities, statistically significant differences were observed when comparing the BCG and WCG, as shown in Tables 5, 6. In the HG test, all three forms (HGsum, HGsum BM, and HGsum height) showed statistically significant differences between the BCG and WCG (*p* = 0.002; *p* = 0.031; and *p* = < 0.001), as well as in the flexibility test (*p* = 0.014) and in the RT evaluation test for both total execution time (*p* = < 0.001) and average time (*p* = < 0.001). A further analysis was carried out between the anthropometric data and coordinative and conditional abilities, and an inversely proportional correlation was found between RT parameters and height. A possible explanation could be that the taller participants had an advantage due to the fixed position of the instrument.

**Table 1:** Total number of the participants divided by sex and occupational position (OP).

Sex	OP	Total number	% of Total
M	BCG	283	46%
	MNG	57	10%
	WCG	188	31%
F	BCG	20	3%
	MNG	6	1%
	WCG	51	9%

The table presents the number of workers as a percentage of the total sample.

**Table 2:** Anthropometric data and qualitative BMI values of the total number of participants divided by sex and occupational position (OP).

					*Shapiro–Wilk*	
	Sex	OP	Mean	SD	*p*	
Age (y)	M	BCG	37.9	9.23	0.002	
		MNG	43.6	7.68	0.072	
		WCG	35.4	7.56	<0.001	
	F	BCG	41.5	7.7	0.068	
		MNG	41.2	8.28	0.153	
		WCG	34.6	8.66	<0.001	
Height (cm)	M	BCG	174.1	6.44	0.300	
		MNG	177.4	5.91	0.176	
		WCG	177.7	6.81	0.032	
	F	BCG	163.7	7.99	0.284	
		MNG	163.7	8.03	0.803	
		WCG	163.6	8.05	0.805	
					Qualitative value	
BMI (kg/m^2^)	M	BCG^b^	25.2	3.44	Overweight	<0.001
		MNG	25.3	2.91	Overweight	0.413
		WCG^b^	23.6	3.42	Normal weight	0.543
	F	BCG	23.7	3.86	Normal weight	0.286
		MNG	23.6	3.8	Normal weight	0.195
		WCG	22.6	3.71	Normal weight	<0.001

Overweight (25–29.9, g/m^2^) and normal weight (18.5–24.9, kg/m^2^), with respective ranges. The letter ‘’b‘’ indicates the comparison between BCG and WCG, indicating statistically significant difference.

**Table 3:** Percentage of weekly physical activity (Weekly PA%) of all participants, reported as absolute values and percentages compared with the OP group and the percentage of specific physical activity (PA%) divided by sex and occupational position (OP).

OP	Sex	n°	Weekly PA (%)	Activity	PA (%)
BCG					
	M	283	48	Aerobic	23
				Power	24
				Sedentary	53
	F	20	55	Aerobic	20
				Power	35
				Sedentary	45
MNG					
	M	57	58	Aerobic	35
				Power	23
				Sedentary	42
	F	6	83	Aerobic	83
				Power	–
				Sedentary	17
WCG					
	M	188	64	Aerobic	35
				Power	29
				Sedentary	36
	F	51	55	Aerobic	27
				Power	28
				Sedentary	45

Aerobic (activities such as running, swimming, cycling, and futsal), Power (gym), and Sedentary (sedentary lifestyle).

**Table 6:** Reaction time total time and reaction time average intermedium results (a sample of 231 participants).

				*Shapiro–Wilk*
	OP	Mean	SD	*p*
RT-TT(s)	BCG^b^	49.5	4.43	< 0.001
	MNG^c^	49.8	2.59	0.279
	WCG^b,c^	47.6	3.53	0.006
RT-AI(ms)	BCG^b^	0.7	0.11	< 0.001
	MNG	0.7	0.06	0.967
	WCG^b^	0.6	0.07	0.004

Statistical differences in comparisons between OPs are indicated as follows: a = BCG versus MNG; b = BCG versus WCG; and c = MNG versus WCG.

**Table 4:** Qualitative and quantitative results of the coordinative and conditional ability tests (Male).

Male				*Shapiro–Wilk*	
	OP	Qualitative value	Mean	SD	*p*
HGsum (kgf)	BCG	Regular (85–115 kgf)	96.0	16.07	0.081
	MNG	Regular (85–115 kgf)	97.5	15.03	0.510
	WCG	Regular (85–115 kgf)	95.5	15.03	0.270
HGsum BM (au)	BCG	–	1.3	0.23	0.907
	MNG	–	1.2	0.22	0.160
	WCG	–	1.3	0.21	0.212
HGsum height (kg/cm) (kg/cm)	BCG	–	0.6	0.09	0.157
	MNG	–	0.6	0.08	0.286
	WCG	–	0.5	0.08	0.251
SR (cm)	BCG	Well above average (21–22 cm)	21.3	7.82	<0.001
	MNG	Well above average (21–22 cm)	21.5	7.00	0.516
	WCG	Well above average (21–22 cm)	21.9	8.27	<0.001
RT-TT (s)	BCG^b^	–	50.3	4.37	<0.001
	MNG*^c^*	–	48.9	3.35	0.544
RT-AI (ms)	BCG^b^	–	0.7	0.10	<0.001
	MNG^c^	–	0.6	0.07	0.871
	WCG^b,c^	–	0.6	0.07	0.003

OPs are indicated by the following: ^a^BCG versus MNG; ^b^BCG versus WCG; and ^c^MNG versus WCG. Handgrid sum (HGsum), Handgrip sum normalized by weight (HGsum BM), Handgrip sum normalized by height (HGsum height), Sit-and-reach (SR), Reaction time-total time (RT-TT), Reaction time-average intermedium (RT-AI).

**Table 5:** Qualitative and quantitative results of the coordinative and conditional ability tests (Female).

Female				*Shapiro–Wilk*	
	OP	Qualitative value	Mean	SD	*p*
HGsum (kgf)	BCG	Very Poor (<71 kgf)	55.8	8.76	0.763
	MNG	Very Poor (<71 kgf)	57.7	9.04	0.152
	WCG	Very Poor (<71 kgf)	58.0	10.11	0.160
HGsum BM (au)	BCG	–	1.0	0.20	0.757
	MNG	–	1.0	0.30	0.436
	WCG	–	1.0	0.19	0.045
HGsum height (kg/cm)	BCG	–	0.4	0.05	0.902
	MNG	–	0.4	0.06	0.550
	WCG	–	0.4	0.06	0.041
SR (cm)	BCG	Well above average (21–22 cm)	27.1	7.22	0.989
	MNG	Above average (19–21 cm)	32.1	6.80	0.103
	WCG	Well above average (21–22 cm)	30.5	8.02	0.994
RT-TT (s)	BCG^b^	–	58.2	3.66	0.726
	MNG	–	55.3	7.34	0.920
	WCG	–	52.2	4.78	0.007
RT-AI (ms)	BCG^b^	–	0.8	0.07	0.413
	MNG	–	0.8	0.14	0.892
	WCG	–	0.7	0.10	0.011

OPs are indicated by the following: ^a^BCG versus MNG; ^b^BCG versus WCG; and ^c^MNG versus WCG. Handgrip sum (HGsum), Hand-grip sum normalized by weight (HGsum BM), Hand-grip sum normalized by height (HGsum height), Sit-and-reach (SR), Reaction time total time (RT-TT), Reaction time average intermedium (RT-AI).

Thus, another analysis was conducted, extrapolating a sample of 231 male participants from the total sample. To better understand this correlation, all participants with a height ranging between 172 cm and 180 cm (176 cm ± 4) from the most representative group were selected. Total and average intermedium time are expressed as mean and relative standard deviation, as shown in Table 4, and ranged between 47 s and 50s for the RT-TT and 0.60 ms and 0.70 ms for the RT-AI. The results highlighted that the WCG outperformed the BCG and MNG in the RT-TT test (*p* = 0.003; *p* = 0.037), while no statistical difference emerged between the BCG and MNG (*p* = 0.947). In addition, in the RT-AI test, the only statistically significant difference was observed between the WCG and BCG (*p* = 0.031), with the WCG demonstrating better performance than the BCG.

## 5 Discussion

Prevention is the primary strategy to mitigate health risks among workers, and evaluating the fitness level through functional tests is a valid tool, as is physical activity according to the American College of Sports Medicine guidelines (39, 40). This study aimed to assess workers’ coordinative and conditional abilities using functional tests to characterize their fitness status, which significantly correlates with overall health. The objective was to link these tests to the Italian annual health surveillance examinations (Legislative Decree 81/2008) and provide an indicator representing coordinative and conditional abilities. Integrating these tests with medical evaluations can improve the effectiveness of health assessments in an automotive corporation, a topic that remains relatively unexplored in the existing literature. Several scientific studies confirm that physically demanding jobs increase the risk of musculoskeletal disorders, leading to premature labor market exits and sickness absences. Indeed, the main objective of this study was to identify assessment tests that can characterize a functional profile of workers to estimate his or her physical well-being and mitigate possible risks in the workplace. This goal aligns with the Total Worker Health® (TWH) concept, which combines protection from work-related safety and health risks with efforts to prevent injury and illness to improve worker well-being. The TWH approach, as explained by Tamers et al. (41), recognizes that workers’ health is affected by work and non-work factors and promotes interventions that address both. Identifying physical labor and workload as critical risk factors for workers’ health and well-being highlights the significance of this study. In addition, physically demanding work can result in work-related fatigue, reducing engagement in leisure-time physical activity and increasing the risk of work-related injuries (42–44). Hence, promoting extra-workplace physical activity is beneficial compared to intense, physically demanding work. Analyzing the results from functional tests on coordinative and conditional abilities, along with anthropometric measures, provides valuable insights (45, 46). Unfortunately, due to data sensitivity, gender distribution among different occupational positions remains undisclosed, especially for BCG workers. Nonetheless, the sample results align with those of the general population for the handgrip (34, 35) and the sit-and-reach tests (36). The main implications of this study are its essential contribution to identifying the difference in physical activity levels among three groups of automotive workers. The examined sample was mostly represented by the BCG, which accounted for more than 50%. In terms of the female participants, more than 65% were from the WCG. The results indicated that the BCG’s physical activity level showed regular upper-limb strength but low trunk flexibility and reaction time performance. Moreover, although they cannot be considered physically inactive, the occupational task of the BCG indicated sedentary behavior. While the characteristics of the WCG and MNG that involve the occupational task, such as sitting posture and upright standing, also indicated sedentary behavior at work, the WCG had the highest proportion of participants engaging in extra-workplace physical activity (about 61%). The group showed better results in the flexibility and reaction time tests. Furthermore, the WCG also showed the best average score regarding the BMI, indicating a balanced body composition. Indeed, the frequency and specific type of physical activity may have contributed to a positive change in the coordinative and conditional abilities. The results of the BCG could be explained by the fact that most movements in the workplace are performed in isometric positions, where the whole-body system is contracted. Therefore, more attention needs to be paid to activities aimed at stretching, which helps make the body more flexible. The BCG had the lowest frequency percentage for extra-workplace physical activity, but the best average results in the upper limb strength test and the worst average results in the flexibility and reaction time tests. The BCG tends to perform better in strength than white-collar workers because their work is almost always more physically demanding. Commonly, the BCG engages in an occupation that requires them to lift, pull, and adjust heavy objects daily. All of this leads this category of workers to perform functional strength training without knowing. The MNG showed a physical activity frequency percentage very close to that of the WCG, along with better strength levels in the proper upper limb strength tests compared to the WCG, as well as better flexibility and reaction times than the BCG. To develop even greater strength, it is advisable to follow an exercise program outside of work as well. Even if a worker is already strong, a strength program outside of work can perfectly complement their job. In addition, it can prevent injuries by counterbalancing the lifting done at work and further strengthening the core muscles. If you work a lot during the day, you need a shorter workout compared to employees who burn a few calories during the day. However, a good stretching program, a light to moderate weightlifting regimen, and some time on a cardio machine a couple of times a week can improve strength. The study emphasizes the importance of incorporating functional tests into the health surveillance examination process Bushman (47). The paper emphasizes non-communicable diseases, highlighting the need for preventive measures to reduce healthcare costs. Presenteeism is also discussed, underscoring the significance of promoting a healthy lifestyle and preventive strategies to maintain workforce productivity and well-being (48, 49). In conclusion, evaluating coordinative and conditional abilities through functional tests is crucial for sustainable prevention. Employers and workers can prevent presenteeism and enhance productivity by identifying weaknesses and promoting a proactive approach to health (50). Emphasizing preventive healthcare measures is a cost-effective and sustainable way to combat the rise of chronic diseases (51). Fostering a health and wellness culture and targeted training programs can lead to a healthier and more productive workforce.

## 6 Limitations and future directions

The study has certain limitations. The first limitation is the low percentage of female workers, particularly in management and consulting roles. This situation mirrors the overall proportion of female workers employed in a factory with various operational positions, a common scenario in Italy. Another limitation of our study is related to the ReaxLights test. The satellite lights were non-movable, making the position unmodifiable based on the height of the participants. Therefore, movable satellites that can adjust according to the height of participants are recommended, along with producing the necessary software and hardware. Future studies should aim to establish a percentiles comparison table or identify the correction factor. It is also suggested an investigation be conducted on energy expenditure and body composition in relation to possible extra-workplace physical activity or on the use of devices designed to assess energy expenditure indirectly. This investigation is important to determine whether workers engage in extra-workplace physical activity before recommending it in the workplace. All these actions can be supported by qualified physical activity professionals who, based on the medical evaluation provided by the occupational physician, can implement prescribed physical activity. In this way, we suggest tailored physical activity programs and functional tests to measure presenteeism and promote a good health status and psycho-physical well-being of workers within an automotive corporation context, integrated into the daily work hours spent in the workplace. While confirming the results of the functional tests and anthropometric measures, we hypothesized that those who spend most of their work time while walking or standing (e.g., blue-collar workers) are not living in good health conditions due to the physical overload caused by their jobs and the lack of time available for engaging in regular physical activity in the workplace context. This condition is less pronounced for managers and white-collar workers, likely due to reduced psycho-physical stress induced by the workplace and the positive effects resulting from morphological and functional adaptations related to regular physical activity practice. Therefore, we further propose using conditional and coordinative abilities tests to support periodic medical check-ups in assessing physical fitness related to wellness and, indirectly, health status. Furthermore, it might be useful to consider the reaction time test as an accurate indicator of wellness, especially when integrated with the hand grip test. This combination could offer a more comprehensive assessment of well-being, particularly for OP-dependent motor tasks.

## Data Availability

The raw data supporting the conclusions of this article will be made available by the authors, without undue reservation.
